# HIV-1 Promotes Intake of *Leishmania* Parasites by Enhancing Phosphatidylserine-Mediated, CD91/LRP-1-Dependent Phagocytosis in Human Macrophages

**DOI:** 10.1371/journal.pone.0032761

**Published:** 2012-03-06

**Authors:** Robert Lodge, Michel Ouellet, Corinne Barat, Guadalupe Andreani, Pranav Kumar, Michel J. Tremblay

**Affiliations:** 1 Centre de Recherche en Infectiologie, Centre Hospitalier Universitaire de Québec - CHUL, Université Laval, Québec, Canada; 2 Département de Microbiologie-Infectiologie et Immunologie, Faculté de Médecine, Université Laval, Québec, Canada; University of California San Francisco, United States of America

## Abstract

Over the past decade, the number of reported human immunodeficiency virus type-1 (HIV-1)/*Leishmania* co-infections has risen dramatically, particularly in regions where both diseases are endemic. Although it is known that HIV-1 infection leads to an increase in susceptibility to *Leishmania* infection and leishmaniasis relapse, little remains known on how HIV-1 contributes to *Leishmania* parasitaemia. Both pathogens infect human macrophages, and the intracellular growth of *Leishmania* is increased by HIV-1 in co-infected cultures. We now report that uninfected bystander cells, not macrophages productively infected with HIV-1, account for enhanced phagocytosis and higher multiplication of *Leishmania* parasites. This effect can be driven by HIV-1 Tat protein and transforming growth factor-beta (TGF-β). Furthermore, we show for the first time that HIV-1 infection increases surface expression of phosphatidylserine receptor CD91/LRP-1 on human macrophages, thereby leading to a *Leishmania* uptake by uninfected bystander cells in HIV-1-infected macrophage populations. The more important internalization of parasites is due to interactions between the scavenger receptor CD91/LRP-1 and phosphatidylserine residues exposed at the surface of *Leishmania*. We determined also that enhanced CD91/LRP-1 surface expression occurs rapidly following HIV-1 infection, and is triggered by the activation of extracellular TGF-β. Thus, these results establish an intricate link between HIV-1 infection, Tat, surface CD91/LRP-1, TGF-β, and enhanced *Leishmania* phosphatidylserine-mediated phagocytosis.

## Introduction

The continuing expansion of the AIDS pandemic has resulted in the establishment of new opportunistic diseases which take advantage of the immunocompromised state prevailing in individuals infected with human immunodeficiency virus type-1 (HIV-1) [1]–[3]. Of these newly recognized opportunistic pathogens, *Leishmania* has risen to considerable importance over the past decade, in large part due to the increased urbanization of (and HIV-1 access to) rural regions in developing countries, and the co-transmission of this protozoan parasite and HIV-1 through the use of contaminated seringes by intravenous drug users [1], [2]. If HIV-1 has ultimately altered the medical, biological and pharmacological aspects of leishmaniasis, *Leishmania* exacerbates HIV-1 infection, complicating treatment and diminishing life expectancy of AIDS patients [1], [2].

*Leishmania* causes several tropical diseases in humans, of which one, visceral leishmaniasis (VL), is potentially fatal. Indeed, over 300,000 new cases of VL are reported annually worldwide, and it is considered to be a major health concern in several developing countries [1]. VL is caused by either one of the closely related species, i.e. *Leishmania donovani* (*L. donovani*), *L. infantum* or *L. chagasi*, and is characterized by hepato-splenomegaly and a massive infection of the reticoendothelial system. The biphasic life cycle of the parasite alternates between motile promastigotes, which are transmitted by the bite of infected sandflies, and the non-motile amastigotes, the major form of the parasite found in humans, which replicate in intracellular phagosomes of infected monocytes and macrophages.

HIV-1 also infects cells of the monocyte lineage (i.e. macrophages and dendritic cells), which act as potential reservoirs for viral replication [4], [5]. It has been shown that both *Leishmania* and HIV-1 greatly impact on macrophage functions, and particularly influence immune response and cytokine production. For example, HIV-1 infection of monocyte-derived macrophages (MDMs) *in vitro* generally inhibits phagocytosis [6]–[9] and elicits a cytokine response leading to secretion of tumor necrosis factor-alpha (TNF-α), interleukin-1 (IL-1), IL-6 and IL-8, among others [10]. The presence of these cytokines greatly impacts on *Leishmania* infection and multiplication in the case of co-infections [1]. In this regard, it has been reported that HIV-1 infection augments *L. infantum* multiplication in MDMs [11], and that, conversely, *Leishmania* enhances HIV-1 replication through inducing the production of TNF-α and IL-1α [12], [13]. Interestingly, HIV-1 augments intake and replication of *Leishmania* in MDMs [11], which is unusual given that, as mentioned above, HIV-1 infection has been shown to inhibit phagocytosis [6]–[8]. Finally, Barreto de Souza and colleagues found that the HIV-1 transactivating protein Tat, through the expression of cyclooxygenase-2, prostaglandin E2 synthesis and the effect of transforming growth factor-beta (TGF-β), mediates increased *Leishmania* replication in co-infected macrophage populations [14]. More recently similar observations were found with the non-pathogenic trypanosomatid *Blastocrithidia culicis* [15].

The precise mechanism(s) by which HIV-1 augments engulfment and replication of *Leishmania* parasites in macrophages, still remains poorly understood. Although we herein confirmed the previously reported enhancing effect of Tat and TGF-β on parasite internalization by MDMs [14], we also found that uninfected bystander macrophages respond to soluble factors secreted by their HIV-1-infected cellular counterparts, and that the former cell population greatly accounts for the reported enhancing effect on *Leishmania* internalization [14]. Furthermore, we demonstrate that uninfected bystander macrophages, through HIV-1-mediated induction of greater surface expression of CD91/LRP-1 (the low density lipoprotein receptor-related protein 1) more actively bind phosphatidylserines located at the surface of the parasite, eventually leading to a superior *Leishmania* entry and replication in the uninfected bystander macrophage subpopulation.

## Materials and Methods

### Establishment and culture of MDMs

Human peripheral blood mononuclear cells (PBMCs) were obtained from healthy blood donors, in accordance with the guidelines of the Bioethics Committee of the CHUL Research Center, by density-gradient centrifugation on Ficoll-Hypaque (Wisent, St-Bruno, QC). All blood donors were informed and agreed to a written consent prior to donating blood. Monocytes were purified by adherence in RPMI-1640 medium (Wisent) supplemented with 5% decomplemented autologous human serum, and allowed to differentiate into MDMs for 6–7 days in RPMI-1640 medium supplemented with 5% autologous human serum and human recombinant macrophage colony-stimulating factor (100 ng/ml, Genscript, Piscataway, NJ). MDMs were harvested by addition of Accutase (eBioscience, San Diego, CA) followed by gentle scraping, and transferred to 6-well or 24-well plates containing glass coverslides (12 mm round, thickness #1, Fisher Scientific, Nepean, ON), at 24 hours prior to HIV-1 infection or cytokine treatments. Following transfer to 6- or 24-well plates, MDMs were cultured in RPMI-1640 supplemented with 5% autologous human serum.

### HIV-1 production and MDM infection

The infectious molecular clone NL4-3-Bal-IRES-HSA was recently described [16]. Briefly, besides encoding all HIV-1 proteins and producing R5 (Bal)-tropic HIV-1 virions, this NL4-3-based vector additionally codes for the cell surface murine heat-stable antigen (HSA)/CD24, enabling for efficient early identification of productively infected cells. Fully competent NL4-3-Bal-IRES-HSA viruses were produced by transient expression in calcium-phosphate transfected 293T cells, and stocks quantitated using an in-house ELISA assay specific for major capsid protein p24 as previously described [17]. Viral preparations underwent a single freeze-thaw cycle before use. MDMs (5×10^5^/well in 6-well plates, 5×10^4^/well in 24-well plates) were infected with NL4-3-Bal-IRES-HSA virus (10 ng of p24/10^5^ cells) for 2 hours, washed extensively with media to remove unadsorbed virions, and cultured for 6 days (unless otherwise indicated) before contact with *L. infantum* amastigotes or zymosan particles. In some experiments, 6-day old virus-infected or uninfected MDM supernatants were harvested, filtered through a 0.22 µm-pore size cellulose acetate membrane (Millipore, Bedford, MA), treated with Efavirenz (EFZ), a non-nucleoside HIV-1 reverse transcriptase inhibitor (50 nM, from the Division of AIDS, NIAID, NIH, through the NIH AIDS Repository Reagent Program, Germantown, MD) and stored at −80°C until use.

### Cytokines, Tat protein, and binding assays for LRPAP/RAP and annexin V phosphatidylserine

HIV-1 Tat protein (used at a final concentration of 100 ng/ml, from Dr. John Brady and DAIDS, NIAID) and rabbit anti-Tat antiserum (used at a dilution of 1∶500, from Dr. Bryan Cullen) and Maraviroc (MVC, used at 50 nM) were obtained through the NIH AIDS Repository Reagent Program. Tat protein was reconstituted in phosphate-buffered saline (PBS) containing 1 mg/ml bovine serum albumin (BSA, Sigma) and 0.1 mM dithiothreitol (DTT). IL-10 and TGF-β (both from Peprotec, Rocky Hill, NJ) were used at concentrations of 10 ng/ml and 2 ng/ml, respectively. The mouse anti-TGF-β neutralizing antibody MAB240 (R&D Systems, Minneapolis, MN) was used at a final concentration of 10 µg/ml. In some TGF-β inhibition assays, type I Furin inhibitor was used at a final concentration of 50 µM (EMD Biosciences, San Diego, CA) and X-VIVO 20 (Lonza BioWhittaker, Walkersville, MD) media was used in some cases. To inhibit phosphatidylserine-mediated phagocytosis, annexin V (BioVision, Mountain View, CA) was added to *Leishmania* parasites or zymosan particles in annexin V-binding buffer (100 mM HEPES, 150 mM NaCl, 5 mM KCl, 5 mM CaCl_2_ and 1 mM MgCl_2_ [pH 7.4]) to the indicated final concentrations for 30 min, prior to phagocytosis. To inhibit CD91/LRP-1-mediated phagocytosis, MDMs were treated with 2 µM LRPAP/RAP (R&D Systems) prior and during phagocytosis of *Leishmania* parasites, or control zymosan particles, for 4 hours.

### *Leishmania* parasites, zymosan particles and phagocytosis assays

Non-transfected or green fluorescent protein (GFP)-expressing *L. infantum* axenic amastigotes transfected with pNEO-GFP (kindly provided by Dr. B. Papadopoulou) [11], were maintained in MAA/20 medium at 37°C in a 5% CO_2_ incubator. MAA/20 consists of modified 199 medium (Gibco/Invitrogen) with Hank's salts, supplemented with 0.5% soybean trypto-casein (Pasteur Diagnostics, Marne la Coquette, France), 15 nM D-glucose, 5 mM L-glutamine, 4 mM NaHCO_3_, 0.023 mM bovine haemin, 25 mM HEPES (at a final pH of 6.5) and 20% fetal bovine serum.

Prior to phagocytosis assays, the concentration of *Leishmania* parasites was determined using a hemocytometer, and parasites were added to MDMs seeded on coverslides at a 5∶1 ratio (10∶1 in the case of the annexin V phosphatidylserine-binding assay). Alternately, Alexa488-labeled zymosan particles (Molecular Probes/Invitrogen, Burlington, ON) were opsonized in RPMI-1640 medium supplemented with 10% complement C5-depleted human serum (C1163, Sigma, St-Louis, MO), and added to MDMs (5 particles/cell, as previously determined by microscopy analysis of serial dilutions of zymosan). After 1 hour, excess targets (i.e. *Leishmania* parasites or zymosan) were washed out, and macrophages were cultured for an additional 3 hours to ensure full phagocytosis of targets. Cells were then fixed in 4% paraformaldehyde (Sigma), washed in PBS and processed for immunofluorescence staining.

### Immunofluorescence staining and microscopy

Following fixation in 4% paraformaldehyde, MDMs on converslides were permeabilized with 0.1% (v/v) Triton X-100 (Sigma) and non-specific binding sites blocked with 1% (w/v) BSA, 10% (v/v) of a pool of decomplemented human sera from several donors and 20% (v/v) normal goat serum (Jackson ImmunoResearch/Cedarlane, West Grove, PA). In the case of HIV-1-infected MDMs, cells were then stained with rat anti-HSA (1∶300 dilution, clone M1/69, BD Biosciences, Mississauga, ON), washed in PBS, and further stained with mouse anti-rat IgG conjugated to Alexa555 (1∶500, Molecular Probes/Invitrogen) and the DNA probe DRAQ5 (1∶1000, Biostatus, Leicestershire, UK). Alternatively, MDMs were stained with Alexa555-conjugated phalloidin (Molecular Probes/Invitrogen) and/or DRAQ5. Stained cells were then mounted using Fluoromount G (Southern Biotech/InterScience, Birmingham, AL) and sealed.

The amounts of internalized *Leishmania* parasites or zymosan particles in MDMs were determined by confocal scanning microscopy, using an Olympus FluoView FV300 microscope (Olympus, Markham, ON) equipped with the appropriate lasers and filters. Further digital imaging was performed using Adobe Photoshop software (v. 6.0, Adobe Systems, San Jose, CA) and NIH ImageJ 1.38, and careful image analysis was done to ensure that all counted targets were fully internalized.

### Flow cytometry analysis of CD91/LRP-1 surface expression

MDMs were harvested by gentle scraping after incubating 10 min at 37°C in PBS containing 5 mM EDTA, washing and fixing in 4% paraformaldehyde. Non-specific binding sites were then blocked with 1% (w/v) BSA, 10% (v/v) of a pool of decomplemented human sera from several donors and 20% (v/v) normal goat serum. Surface expression of CD91/LRP-1 was determined using FITC-labeled mouse anti-human CD91 antibodies (1∶100; BD Biosciences/Pharmingen). Controls consisted of cells labeled with an isotype-matched FITC-tagged antibody (IgG1κ). Cells were analyzed using a Coulter EPICS XL flow cytometer (Beckman-Coulter, Miami, FL) and FCS express software. A minor displacement of control curves were observed in HIV-1-infected MDMs (as compared to uninfected cells), and was taken into account.

### Statistical analysis

The total number of GFP-tagged *Leishmania* parasites or fluorescent-labeled zymosan particles per 100 MDMs (i.e. productively infected with HIV-1/HSA-positive, uninfected bystander/HSA-negative and mock-infected control) were determined. Results presented are expressed as means ± standard error of the mean (SEM) of triplicate samples. Results shown are of experiments from cells derived from one donor representative of at least three different healthy blood donors. Analyses were performed using Student's two-sample one (when mentioned) or two-tail unequal variance tests. *P* values of <0.05 were deemed statistically significant.

## Results

### HIV-1 infection augments internalization and survival of *Leishmania* parasites in uninfected bystander MDMs

It has been established that HIV-1 infection markedly enhances *Leishmania* proliferation in MDMs [11], [14]. However, it is still unclear whether the HIV-1-mediated enhancement of parasite growth occurs in MDMs productively infected with HIV-1 and/or uninfected bystanders cells (i.e. uninfected cells residing in a population also containing virus-infected cells). It was therefore of high interest to investigate and compare the entry and survival of *Leishmania* parasites in both uninfected bystander and HIV-1-producing macrophage subpopulations. To this end, we used a fully competent R5-tropic, HSA-encoding viral construct to discriminate between both populations, by the expression of the HSA reporter cell surface molecule. Moreover, studies were all performed with *L. infantum* amastigotes because it is the *Leishmania* strain most frequently diagnosed in co-infected individuals and the amastigote form is considered as the developmental stage of the parasite that is responsible for maintaining and spreading the parasitic infection in humans.

**Figure 1:**
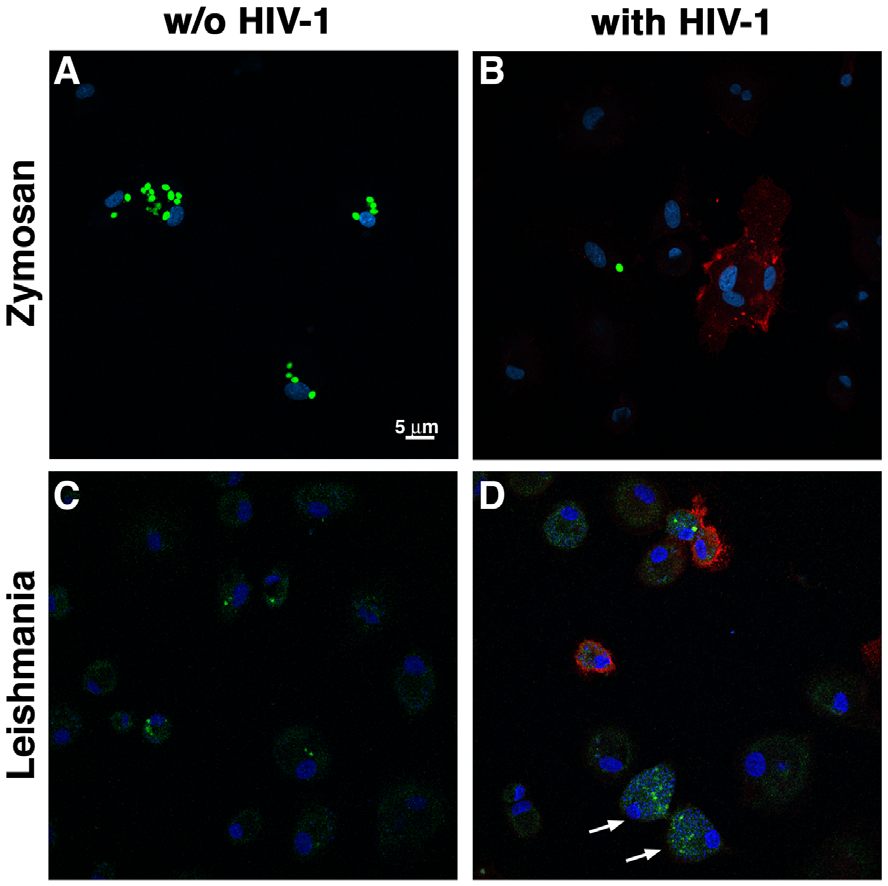
HIV-1 infection exerts a different effect on MDM phagocytosis of zymosan particles or *Leishmania* parasites. Mock-infected control MDMs (panels A and C) or cells infected for 6 days with NL4-3-Bal-IRES-HSA reporter virus (panels B and D) were put in contact either with complement-opsonized Alexa488-tagged zymosan particles (panels A and B) or GFP-expressing *Leishmania* parasites (panels C and D) (both shown in *green*) for 1 hour. Next, excess zymosan particles/*Leishmania* amastigotes were washed out and MDMs cultured for an additional 3 hours. Cells were then fixed, mounted and immunostained for HSA (shown in *red*) and DNA (using DRAQ5, shown in *blue*) to detect HIV-1-infected cells and *Leishmania* DNA/host cell DNA, respectively. Shown are representative images obtained by confocal microscopy. Arrows indicate uninfected bystander MDMs displaying numerous internalized *Leishmania* parasites.

**Figure 2:**
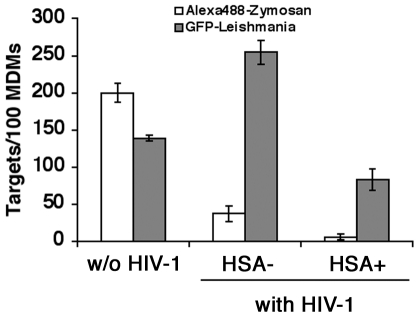
*Leishmania* parasites are internalized more efficiently in uninfected bystander MDMs compared to virus-infected cells. The total numbers of complement-opsonized Alexa488-labeled zymosan particles or GFP-expressing *Leishmania* parasites internalized were determined in mock-infected control, uninfected bystander, or productively HIV-1-infected MDMs, by fluorescence microscopy, as previously illustrated in Figure 1. Results shown are of a single representative donor out of a grand total of nine donors (mean number of targets ± SEM).

Following 6 days of HIV-1 infection, we observed, using fluorescence confocal microscopy, that between 7 and 12% of MDMs (compiled from 9 independent donors) were productively infected with HIV-1 (i.e. HSA+) (data not shown). We then compared the capability of productively-infected and uninfected bystander MDMs, along with mock-infected control cells, to internalize GFP-expressing *Leishmania* parasites. We also assessed the phagocytic index of zymosan using complement-opsonized Alexa488-labeled particles based on the notion that such protein-carbohydrate complexes prepared from yeast cell wall are commonly used targets in phagocytosis assays. Representative confocal microscopy images of mock-infected (i.e. without HIV-1) (left panels) or MDMs inoculated with HIV-1 (i.e. both uninfected bystander/HSA- and productively-infected cells/HSA+) (right panels) are shown in Figure 1. As expected, quantitative analyses of confocal microscopy images indicated that mock-infected control cells internalized much higher amounts of zymosan particles than uninfected bystander (i.e. HSA−) (P = 0.006) and HIV-1-infected MDMs (i.e. HSA+) (P = 0.017) (Figure 2). Moreover, zymosan internalization was more efficient in uninfected bystander cells compared to HIV-1-infected MDMs. On the other hand, uninfected bystander MDMs exhibited a significant increase in *Leishmania* phagocytosis, as compared to either their HSA-expressing counterparts (P = 0.008) or the mock-infected control MDMs (P = 0.049). Therefore, opposite effects were observed in uninfected bystander MDMs compared to mock-infected control MDMs concerning the engulfment of opsonized zymosan particles (i.e. decrease) or the phagocytosis of *Leishmania* parasites (i.e. increase).

### MDMs productively infected with HIV-1 secrete a soluble factor that enhances *Leishmania* uptake in uninfected bystander cells

**Figure 3:**
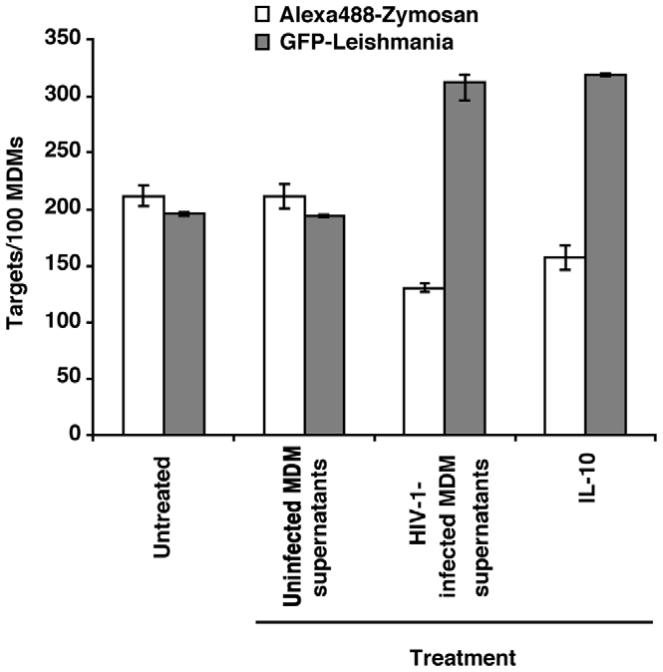
MDMs productively infected with HIV-1 secrete a factor that is necessary for inducing enhanced *Leishmania* parasite intake. MDMs were treated for 24 hours with cell-free supernatants from 6-day old virus-infected macrophages or uninfected MDM supernatants supplemented with EFZ. Mock-treated cells or MDMs treated for 24 hours with IL-10 were used as controls. Next, MDMs were pulsed for 1 hour either with complement-opsonized Alexa488-labeled zymosan particles or GFP-expressing *Leishmania* parasites. Thereafter, excess zymosan particles/*Leishmania* parasites were washed out and MDMs cultured for another 3 hours. After fixing and mounting the cells, the numbers of internalized zymosan particles or *Leishmania* parasites were then determined by fluorescence microscopy. Results are from a donor representative of three (mean number of targets ± SEM).

In order to identify by what mechanism *Leishmania* phagocytosis was specifically enhanced in the uninfected bystander cell subpopulation compared to HIV-1-infected MDMs, we investigated if this effect was dependent on a soluble factor released by productively HIV-1-infected MDMs. We therefore treated a fresh culture of MDMs (i.e. uninfected with HIV-1) with 6-day old cell-free supernatants from virus-infected or uninfected MDMs to which was added Efavirenz (EFZ), a specific inhibitor of the virus-encoded reverse transcriptase enzyme. This enabled us to abrogate viral infection when such supernatants were added to new cultures of MDMs, as detected by ELISA against the major core p24 protein for up to 7 days (data not shown). As depicted in Figure 3, a 24-hour exposure of uninfected MDMs to supernatants from HIV-1-infected macrophages containing EFZ was sufficient to enhance *Leishmania* phagocytosis to more than 50% of those found in untreated control MDMs, but to similar levels found in cells treated with IL-10 (P = 0.67), a cytokine known to favor *Leishmania* multiplication and survival [18]. Additionally, a significant drop in the ability of the supernatant-treated cells to internalize zymosan particles was also observed as compared to untreated cells (P = 0.029). These results suggested that a soluble factor present in cell-free supernatants from HIV-1-infected MDMs was responsible for the higher parasite uptake in uninfected bystander cells.

### Tat and TGF-β secretion enhance parasite entry in MDMs

**Figure 4:**
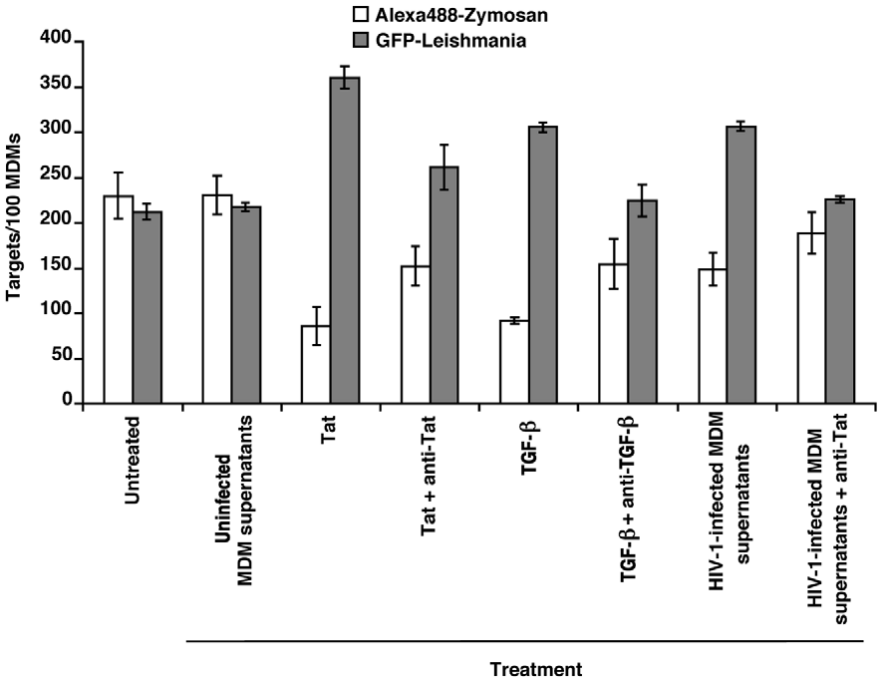
Tat and TGF-β mediate enhanced *Leishmania* parasite intake in MDMs. Cells were either left untreated, or treated for 24 hours with uninfected MDM supernatants, Tat or TGF-β, prior to a 1 hour exposure to Alexa488-labeled zymosan particles or GFP-expressing *Leishmania* parasites. In some cases, anti-Tat, or anti-TGF-β neutralizing antibodies, were added at the time of incubation with either Tat or TGF-β, respectively. Alternatively, anti-Tat was added to supernatants from HIV-1-infected macrophages, and this mix added to MDMs at 24 hours before performing the phagocytosis assay. Thereafter, excess zymosan particles/*Leishmania* parasites were washed out and MDMs cultured for another 3 hours. Numbers of internalized zymosan particles or *Leishmania* parasites were determined as described in Materials and Methods. Results are the means obtained from a donor representative of three (mean number of targets ± SEM).

It has previously been reported that the addition of the HIV-1 early protein Tat to MDM supernatants leads to enhanced internalization of *L. amazonensis* promastigotes [14]. In order to investigate which soluble factor(s) is responsible for the enhanced uptake of *L. infantum* amastigotes in uninfected bystander cells, we added Tat or TGF-β to the supernatants of MDMs for 24 hours prior to addition of fluorescent-tagged parasites or zymosan particles. The use of TGF-β is supported by the previous demonstration that this cytokine is induced by Tat [19] and can increase the survival of intracellular parasites in macrophages [15]. As illustrated in Figure 4, a significant increase in *Leishmania* internalization within MDMs was observed when either Tat (P = 0.0067) or TGF-β (P = 0.016) was added, as compared to untreated control cells or cells treated with uninfected MDM supernatants. This effect was inhibited partially when neutralizing antibodies to Tat or TGF-β were added simultaneously with the corresponding proteins. Furthermore, anti-Tat also inhibited the up-regulatory effect of EFZ-treated supernatants from HIV-1-infected macrophages on the amastigote uptake. These effects were particularly significant with cells that internalized the higher number of parasites (i.e. 5 or more) (data not shown). Internalization of zymosan particles was reduced upon a treatment with either Tat or TGF-β.

### Tat affects phosphatidylserine-mediated phagocytosis

**Figure 5:**
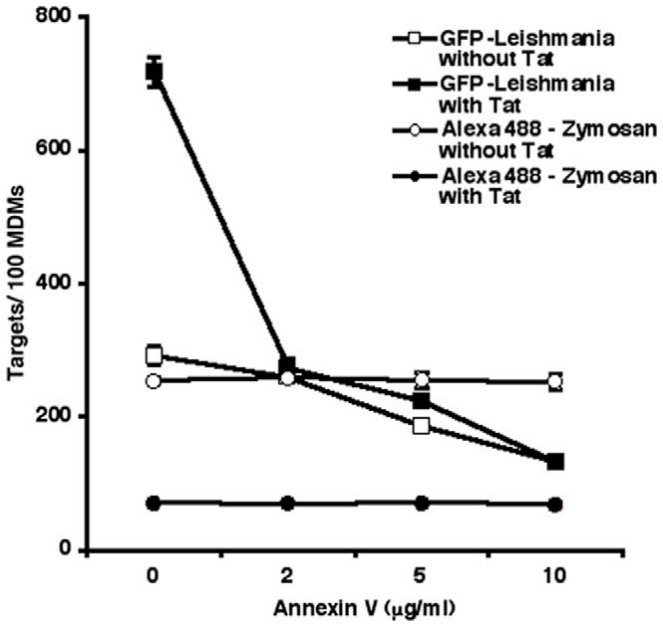
Enhanced intake of *Leishmania* parasites in uninfected bystander MDMs is dependent on a surface macrophage receptor for phosphatidylserine. Alexa488-labeled zymosan particles or GFP-expressing *Leishmania* parasites were first pre-incubated for 30 min in annexin V-binding buffer with the listed concentrations of annexin V, and added to MDMs previously treated or not with Tat. The amount of internalized zymosan particles or *Leishmania* parasites was determined as described in Materials and Methods. Results are the means of a donor representative of three (mean number of targets ± SEM).

We next investigated how the addition of Tat exerts a positive effect on *Leishmania* parasite entry, whereas it can significantly reduce the intake of zymosan particles. Although HIV-1 infection is generally considered to inhibit phagocytosis [6]–[9], it is possible that uptake is enhanced in specific cases involving Tat or TGF-β. Since *Leishmania* amastigotes use phosphatidylserine-mediated phagocytosis to enter phagocytes [20]–[22], we set out to determine if this mechanism is promoted in Tat-treated MDMs. In order to answer this fundamental question, the phosphatidylserine-binding protein annexin V was used to mask this residue at the surface of *Leishmania* amastigotes. The binding of annexin V was specific to amastigotes since this molecule displays no effect with respect to the uptake of complement-opsonized zymosan particles in MDMs (Figure 5). However, as little as 2 µg/ml of annexin V was sufficient to inhibit the Tat-dependent increase in *Leishmania* entry (P<0.0001). Further increasing concentrations of annexin V brought amastigote phagocytosis to levels below that of control macrophages (i.e. untreated with Tat), although at a slower rate of inhibition.

### HIV-1-mediated enhanced amastigote intake is linked to increased levels of CD91/LRP-1

If phosphatidylserine-mediated phagocytosis of *Leishmania* is increased by Tat or TGF-β, then such enhanced entry may be due to greater surface expression of phosphatidylserine-binding receptors on MDMs. We therefore analyzed the surface expression of putative phosphatidylserine receptors such as scavenger receptor CD36 [23], phosphatidylserine receptor (PSR) [24], or brain-specific angiogenesis inhibitor 1 (BAI-1) [25] on virus-infected macrophage populations, or on MDMs treated with TGF-β, Tat, or supernatants from HIV-1-infected cells. We did not find any statistically significant differences in the three studied phosphatidylserine-binding receptors for the untreated or treated MDMs (data not shown).

**Figure 6:**
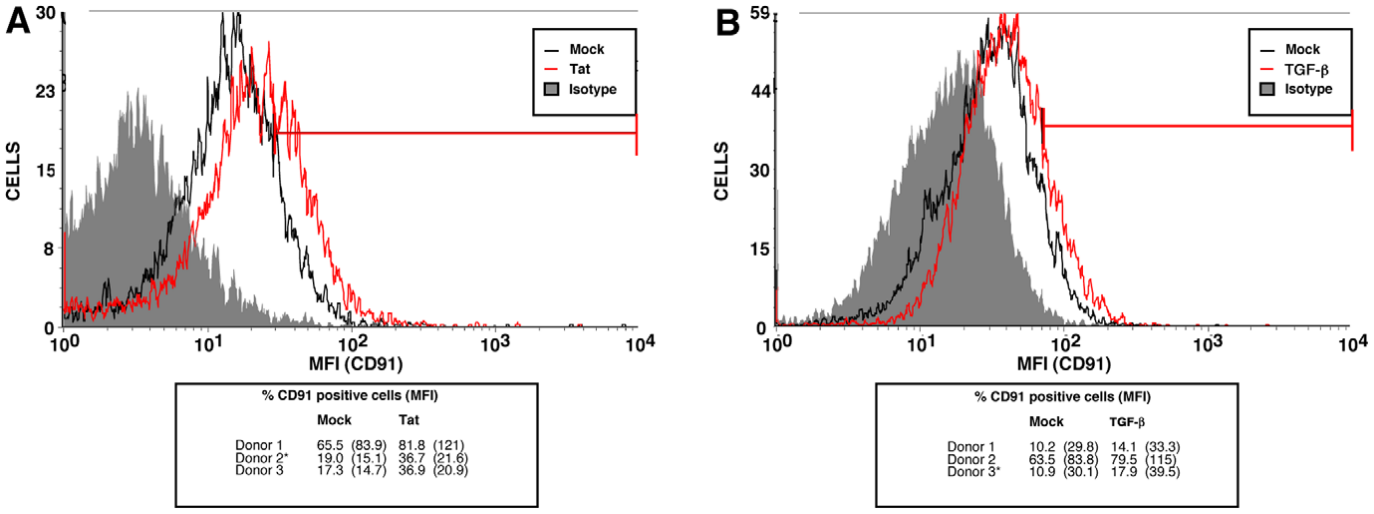
Tat and TGF-β enhance CD91/LRP-1 surface expression in macrophages. MDMs were treated for 24 hours with either Tat (panel A) or TGF-β (panel B), and cell surface CD91/LRP-1 determined by flow cytometry as described in Materials and Methods. Graphs shown are from results obtained from a donor (depicted with an asterisk) chosen from the three reported in the small table below each graph. Different donors were used in panels A and B. The percentage of CD91 positive cells and mean fluorescence intensity (MFI) (shown in parentheses) are illustrated for the six different donors tested.

**Figure 7:**
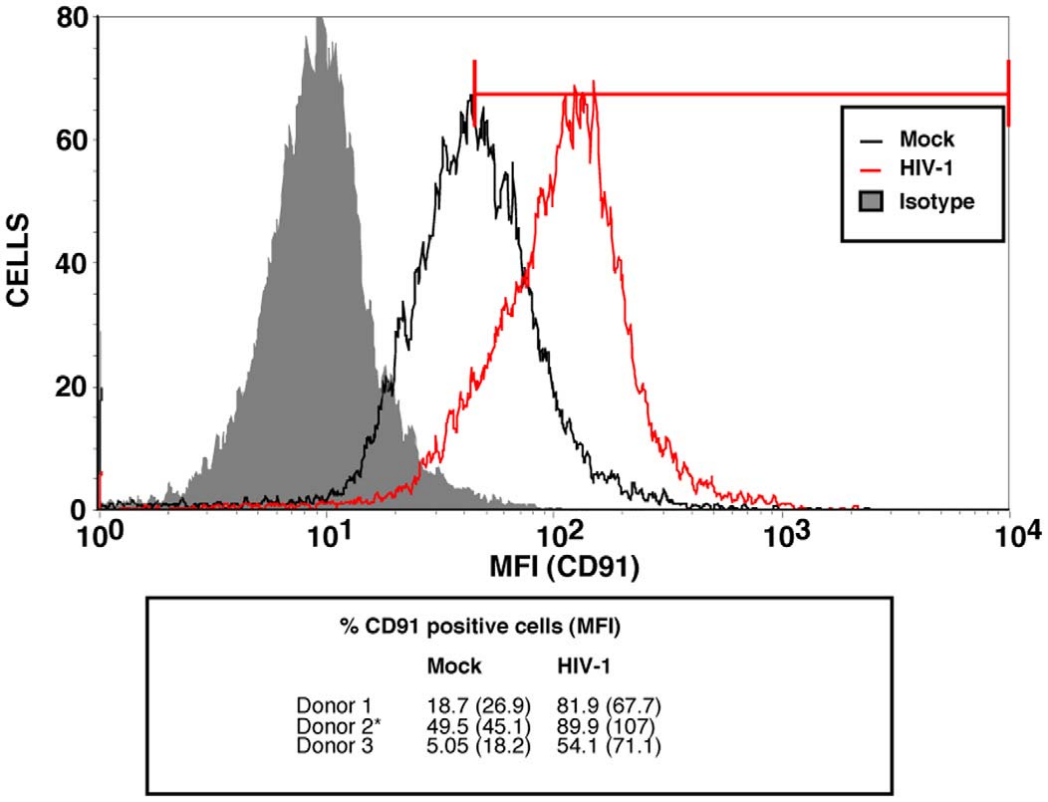
HIV-1 infection enhances CD91/LRP-1 expression in macrophages. MDMs were infected for 6 days with HIV-1, and cell surface expression of CD91/LRP-1 was determined by flow cytometry as described in Materials and Methods. The graphs shown is from results obtained from a donor (depicted with an asterisk) chosen from the three reported in the small table below the graph. The percentage of CD91 positive cells and mean fluorescence intensity (MFI) (shown in parentheses) are illustrated for the three different donors tested.

Given that the cell surface expression of the three tested receptors that directly interact with phosphatidylserines is not enhanced by Tat or TGF-β, we investigated if receptors involved indirectly with phosphatidylserine-mediated phagocytosis, such as CD91/LRP-1, were upregulated in such conditions. As shown in Figure 6, MDMs either exposed to Tat or TGF-β for a brief time period (i.e. 24 hours) showed a slight but reproducible enhancement of surface expression of CD91/LRP-1 when compared to untreated control cells (called mock). However, as illustrated in Figure 7, the increase in surface CD91/LRP-1 was much greater in 6 day old HIV-1-infected MDMs.

**Figure 8:**
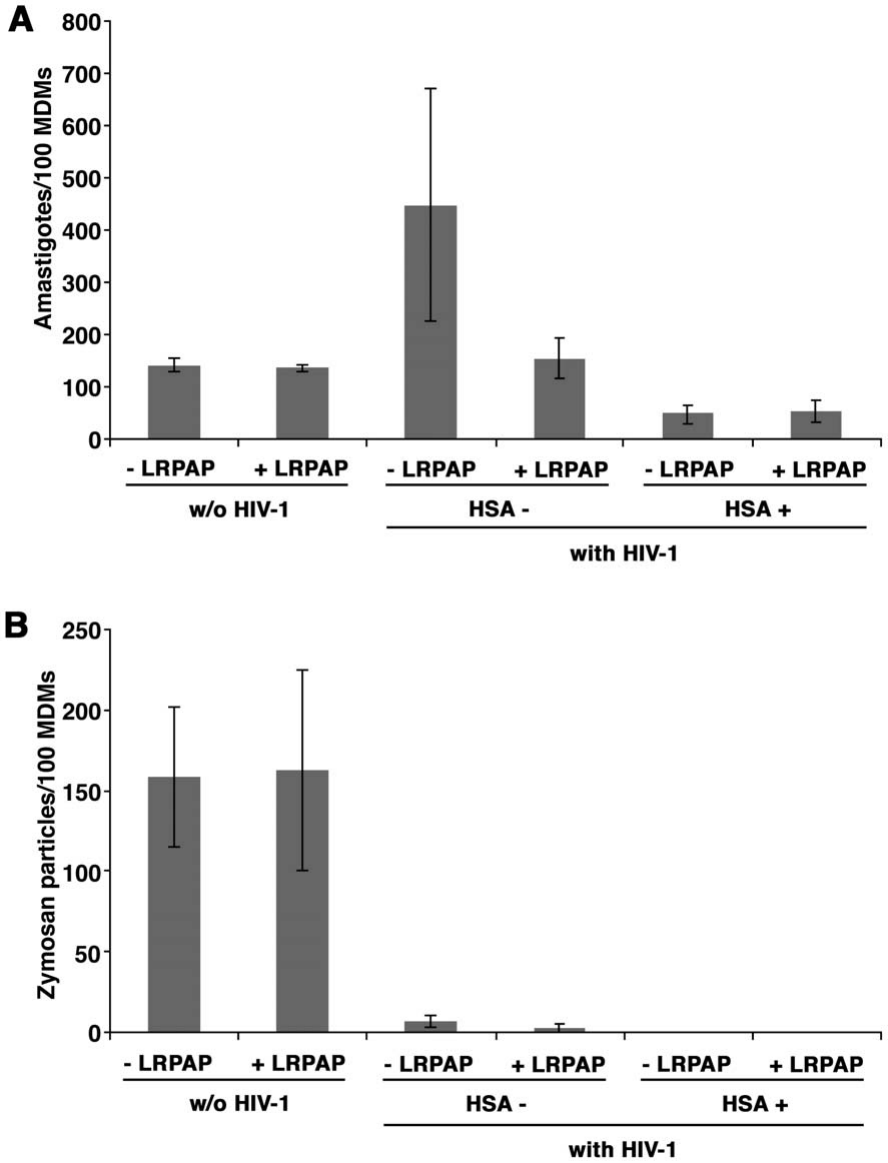
HIV-1-mediated increase in *Leishmania* phagocytosis in uninfected bystander MDMs is inhibited by LRPAP/RAP. Mock-infected or 6 day HIV-1-infected MDMs were treated for 4 hours with the CD91/LRP-1-ligand antagonist, LRPAP/RAP, prior and during phagocytosis of *Leishmania* parasites (panel A) or zymosan particles (panel B), for 4 hours. MDMs were then fixed, stained and mounted for confocal microscopy analysis as described in Materials and Methods. The numbers of internalized targets were then determined. Results shown are the means of four distinct donors ± SEM.

In order to directly demonstrate the contribution of CD91/LRP-1-mediated phagocytosis in the HIV-1-dependent enhancement in *Leishmania* parasite uptake by uninfected bystander MDMs, we analyzed the effect of LRPAP/RAP, an antagonist of CD91/LRP-1-ligand interactions [26], on *Leishmania* or zymosan phagocytosis in HIV-1-infected MDM populations. Mock- or HIV-1-exposed macrophages were therefore treated with LRPAP/RAP prior and during phagocytosis of *Leishmania* parasites or zymosan particles. As shown in Figure 8A, a significant decrease in *Leishmania* parasite internalization was observed in uninfected bystander MDMs in presence of LRPAP/RAP when compared to the untreated counterpart (one tail, P = 0.038). Indeed, addition of the agonist brought down the numbers of internalized parasites in uninfected bystander cells to levels found in uninfected MDMs (P = 0.58). As shown in Figure 8B, the addition of LRPAP/RAP had no effect on zymosan phagocytosis, and thus was specific to CD91/LRP-1-mediated phagocytosis (P = 0.12).

### HIV-1 infection activates TGF-β, which in turn upregulates surface expression of CD91/LRP-1

**Figure 9:**
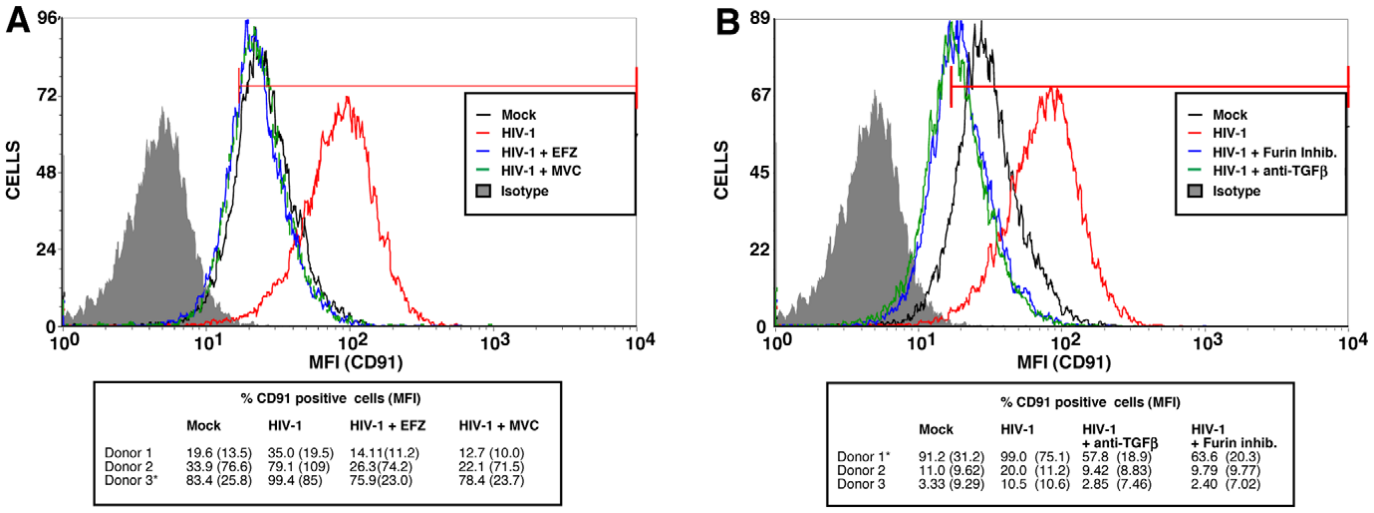
HIV-1 infection of MDMs activates serum TGF-β, leading to enhanced cell surface CD91/LRP-1 expression. HIV-1-infected MDMs were treated either with MVC or EFZ (panel A) or either neutralizing TGF-β antibodies or Furin inhibitor I (panel B) during HIV-1 infection for 8 hours, and cell surface CD91/LRP-1 determined by flow cytometry as described in Materials and Methods. Both uninfected and untreated HIV-1-infected MDMs were used as controls. Graphs shown are from results obtained from a donor (shown with an asterisk) chosen from the three reported in the small table below each graph. Different donors were used in panels A and B. The percentage of CD91 positive cells and mean fluorescence intensity (MFI) (shown in parentheses) are illustrated for the six different donors tested.

Given our previous observations that Tat (and TGF-β) augment surface CD91/LRP-1 on macrophages, on one hand, and that HIV-1 infection enhances *Leishmania* internalization in uninfected bystander MDMs, on the other, we set out to determine if virus infection promotes either TGF-β secretion or activation. Quantitative analysis of newly synthesized TGF-β transcripts in HIV-1-infected MDM populations, as compared to uninfected cells, determined that no significative change in TGF-β transcription was induced by HIV-1 (data not shown). However, given that much TGF-β is secreted in an inactive form [27], [28], and that serum is a rich source of TGF-β [27], we investigated if HIV-1 infection of MDMs led to TGF-β activation in the serum-containing media. This was achieved by comparing the TGF-β-dependent rise of surface CD91/LRP-1 in 8-hour HIV-1-infected macrophages in the presence of Furin inhibitor I, which has been reported to suppress TGF-β activation [29]–[31]. As shown in Figure 9A, MDMs treated prior and during HIV-1 infection with the viral entry inhibitor Maraviroc (MVC), a CCR5 antagonist, or EFZ had similar levels of surface CD91/LRP-1 as compared to uninfected MDMs. This indicates that productive HIV-1 infection is required to modulate surface expression of the scavenger receptor CD91/LRP-1 in MDMs. Interestingly, MDMs treated with either Furin inhibitor I, or neutralizing anti-TGF-β antibodies, at the time and following HIV-1 infection expressed significantly less surface CD91/LRP-1 as compared to untreated cells (Figure 9B). These data suggest that productive HIV-1 infection triggers the release of TGF-β activation factor(s) which, in turn, enhances CD91/LRP-1 surface expression in MDMs.

## Discussion

The growing spread of the HIV-1 pandemic from urban centers to outlying periurban and rural areas in developing countries has given rise to new opportunistic co-infections. Although many of the first *Leishmania*/HIV-1 co-infections were observed in Southern Europe and the Mediterranean basin [1], and were strongly prevalent among intravenous drug users, *Leishmania* has taken advantage of the growing HIV-1 geographical overlap and is now considered as a significant opportunistic infection [1], [2]. In addition, both *Leishmania* and HIV-1 act to each other's benefit in the co-infected individual. Indeed, HIV-1 often reinitiates or worsens *Leishmania* infections, whereas the presence of the protozoan parasite accelerates the progression towards AIDS [1], [2].

Both *Leishmania* and HIV-1 infect cells of the macrophage/monocyte lineage. Furthermore, in co-infected cultures, the two pathogens enhance their counterpart's multiplication by inducing an array of cytokines. For instance, in human primary dendritic cell/CD4^+^ T cell cocultures, *L. infantum* amastigotes enhance HIV-1 production by inducing IL-6 and TNF-α [32]. Using human macrophages, we and others have reported that *Leishmania* enhances HIV-1 replication in this cell type by the release of the proinflammatory cytokines IL-1α and TNF-α [13]. On the other hand, HIV-1 infection promotes *Leishmania* survival and uptake by macrophages [11], [14]. The fact that HIV-1 enhances the parasite's uptake sharply contrasts with most observations concerning HIV-1's effect on phagocytosis [6], [7], [8], [9]. Indeed, although HIV-1 infection has been reported to enhance entry of other trypanosomatids, such as *Blastocrithidia culicis* [15], HIV-1 infection has been generally reported to inhibit macrophage functions, including important signal transduction pathways and mechanisms involved in phagocytic uptake of microbes and other targets [7], [9]. In this report, we further investigated the mechanisms that allow for enhanced *Leishmania* uptake in HIV-1-infected macrophages.

Our use of a novel HIV-1 construct encoding for all viral proteins and murine HSA (CD24) allowed for efficient identification of cells productively infected with HIV-1 and the surrounding uninfected bystander cell counterpart. Quantitation of parasite phagocytosis in both subpopulations clearly revealed that uninfected bystander cells greatly account for the HIV-1-dependent enhanced intake of *L. infantum* amastigotes in MDMs. Our observations also suggest that cells productively infected with HIV-1 release soluble factors which, in turn, act on uninfected bystander neighbor cells. Although such factors are potentially involved in higher parasite uptake, they could also account for the loss in phagocytosis of complement-opsonized zymosan particles. This two-way effect on phagocytosis was also observed in MDMs treated with supernatants harvested from HIV-1-infected macrophages, in which virus replication was inactivated by the antiviral compound Efavirenz. This observation again strongly implies that soluble, secreted compounds from HIV-1-infected macrophages are directly responsible for the superior uptake of *Leishmania* parasites by uninfected bystander MDMs.

Barreto-de-Souza and colleagues found that enhanced multiplication of *L. amazonensis* in HIV-1-infected MDM cultures is driven primarily by the release of the viral Tat protein, which in turn induces cyclooxygenase-2 (COX-2) expression [14]. Furthermore, it was also found that neutralization of TGF-β1 reduced the Tat-mediated effect on parasite growth. Tat is a small protein that interacts with the transactivation response element at the 5′-end of viral mRNAs. In addition to this, Tat is released by infected cells, as has been reported in *in vitro* and *in vivo* studies [33], [34]. Thus, the viral protein can be internalized by surrounding neighbor cells, affecting their normal function, inducing apoptosis, or modulating cytokine secretion. Of particular interest, both IL-10 and TGF-β1 induction in macrophages have been associated with the presence of Tat [19], [35]. We found that the addition of either Tat, IL-10, or TGF-β to MDMs is sufficient to both enhance *L. infantum* amastigote entry into macrophages and, on the other hand, inhibit complement-opsonized zymosan phagocytosis. Accordingly, IL-10 and TGF-β have been reported to decrease phagocytic function in macrophages [36], and HIV-1 infection has been reported to inhibit complement receptor-mediated entry [6]. Interestingly, Barreto-de-Souza and co-workers used promastigotes as targets throughout their experiments [14], therefore suggesting that Tat also enhances promastigote survival and differentiation into amastigotes, in addition to parasite entry into macrophages. Using *L. infantum* promastigotes, we also observed elevated entry into virus-infected MDMs populations, and, as with amastigotes, found that uninfected bystander cells had internalized most of the parasites (data not shown). However, given that entry pathways used by promastigotes and amastigotes in macrophages can be significantly different, further studies are needed to address which receptors are involved in the Tat-mediated enhanced uptake of promastigotes in MDMs. Interestingly, it is also possible that at least some promastigotes use phosphatidylserine as a mode of entry into macrophages, as recently reported [37], [38].

Several reports have suggested that *Leishmania* amastigotes, among other parasites, enter phagocytes using phosphatidylserine residues exposed on the parasite surface [22], [39]. This ensures minimal monocyte/macrophage activation, and mimics the intake of apoptotic cells. Furthermore, it has been previously reported that both TGF-β and IL-10 are produced in such anti-inflammatory situations [22], [40]. In addition to these observations, HIV-1 replication is upregulated by the phagocytosis of apoptotic cells [41], [42], therefore suggesting that the resulting anti-inflammatory cytokines may also contribute to viral pathogenesis. Accordingly, we were successful in inhibiting *Leishmania* amastigote entry in MDMs using annexin V. The annexin-V-mediated effect was specific to amastigotes, since complement-opsonized zymosan phagocytosis was not affected. In Tat-treated MDMs, annexin V sharply reversed any Tat-mediated enhanced *Leishmania* internalization, suggesting that Tat's effect on *Leishmania* entry is greatly dependent on the parasite's surface phosphatidylserines. However, the annexin V-mediated decrease in amastigote entry was less efficient at higher concentrations, possibly indicating that other modes of entry are also used once a saturating amount of annexin V has blocked all available phosphatidylserine.

Several macrophage receptors have been implicated in the phagocytic process of apoptotic cells [43]. However, not all the receptors that bind phosphatidylserine have been fully characterized. For instance, a putative receptor named PSR [24], was at first a strong candidate, though it is now clearly dissociated with phosphatidylserine-mediated phagocytosis [43]. The scavenger receptor CD36 [23], and more recently BAI-1 [25], TIM-4 [44], [45] and Stabilin-2 [46] have all been proposed to bind phosphatidylserine residues. However, CD36 and TIM-4 may only be involved in binding or tethering, and not full target engulfment [47]. Given that none of the surface expression of these receptors were upregulated by HIV-1 infection in MDMs, we focused on receptors of phosphatidylserine-binding ligands, such as CD91/LRP-1. CD91/LRP-1 binds β2-glycoprotein, which has been reported to interact with phosphatidylserines of apoptotic bodies [26]. Our findings that surface CD91/LRP-1 is increased in MDMs treated with Tat, as well as following HIV-1 infection, suggested, at first, that this entry pathway is involved in enhanced amastigote uptake by macrophages. This hypothesis was confirmed by the use of the CD91/LRP-1-ligand interaction antagonist LRPAP/RAP, which inhibited the enhanced *Leishmania* entry in HIV-1-infected macrophage populations. Thus, amastigote/β2-glycoprotein complexes are taken in more efficiently in HIV-1-infected MDM populations, having greater access to the CD91/LRP-1 receptor. Even though uninfected bystander MDMs in HIV-1 infection account for *Leishmania* enhanced entry (not the macrophages productively infected with HIV-1), it is possible that both macrophage subpopulations may express higher levels of surface CD91/LRP-1. It is possible that productively HIV-1-infected MDMs are unable to sustain additional *Leishmania* entry/multiplication, given their underlying viral infection. Finally, it is noteworthy that not all phosphatidylserine-mediated phagocytosis is necessarily enhanced by HIV-1 infection, since apoptotic cell phagocytosis may also be inhibited by the virus in certain cases [9].

Although we found that neutralizing anti-TGF-β antibodies were sufficient to counteract the effect of either HIV-1 infection or that of the addition of TGF-β on CD91/LRP-1 surface expression and *Leishmania* entry in MDMs, respectively, it is noteworthy that we were unable to detect, by quantitative RT-PCR, production of newly secreted TGF-β in HIV-1-infected MDM populations. Furthermore, enhanced surface CD91/LRP-1 expression could be detected as early as 8 hours following HIV-1 infection, suggesting that the mechanisms involved were happening early in the virus infection process. Indeed, since Efavirenz efficiently inhibited enhanced amastigote entry, some early active steps in the HIV-1 viral cycle must be linked to extracellular TGF-β activation and CD91/LRP-1 surface expression. Our Furin inhibitor I assays also suggest the existence of such mechanisms. It is therefore conceivable that some host factor(s), in reaction to HIV-1 infection, or even HIV-1 viral factors may lead to TGF-β activation. In this regard, Tat produced prior to HIV-1 integration has been reported in infected cells [48]. However, further investigation into these mechanisms is needed.

In the last few years, a concept derived by analogy to the CD4^+^ T helper cell paradigm (i.e. Th1, Th2 and Th17 effector cells) has been developed based on a polarization of macrophages into opposing pro- and anti-inflammatory programs [36], [49], [50]. In this model, M1 macrophages produce IL-1β and TNF-α, reactive oxygen and nitrogen intermediates, among others, whereas M2 macrophages, which display a higher heterogeneity, secrete IL-10 and express scavenger and mannose receptors. Among M2 cells, the M2c subpopulation is induced primarily by IL-10 and has a role in immune response suppression [36], [50]. Given our current observations on IL-10 and TGF-β, and their impact on *Leishmania*/HIV-1 interactions in macrophages, further investigation into M2c macrophage polarization and possible modulation by HIV-1 Tat may help in the understanding of the complex interactions between these two important human pathogens.
